# Tailored BiVO_4_ Photoanode Hydrophobic Microenvironment Enables Water Oxidative H_2_O_2_ Accumulation

**DOI:** 10.1002/advs.202300169

**Published:** 2023-03-31

**Authors:** Man Ou, Mei Geng, Xiangle Fang, Wenfan Shao, Fenghong Bai, Shipeng Wan, Caichao Ye, Yuping Wu, Yuhui Chen

**Affiliations:** ^1^ School of Energy Science and Engineering Nanjing Tech University Jiangsu 211816 P. R. China; ^2^ Academy for Advanced Interdisciplinary Studies and Guangdong Provincial Key Laboratory of Computational Science and Material Design Southern University of Science and Technology Guangdong 518055 P. R. China; ^3^ Department of Chemical and Biomolecular Engineering Yonsei University Seoul 120749 Republic of Korea

**Keywords:** H_2_O oxidation, H_2_O_2_, hydrophobicity, microenvironment, photoelectricity

## Abstract

Direct photoelectrochemical 2‐electron water oxidation to renewable H_2_O_2_ production on an anode increases the value of solar water splitting. BiVO_4_ has a theoretical thermodynamic activity trend toward highly selective water oxidation H_2_O_2_ formation, but the challenges of competing 4‐electron O_2_ evolution and H_2_O_2_ decomposition reaction need to overcome. The influence of surface microenvironment has never been considered as a possible activity loss factor in the BiVO_4_‐based system. Herein, it is theoretically and experimentally demonstrated that the situ confined O_2_, where coating BiVO_4_ with hydrophobic polymers, can regulate the thermodynamic activity aiming for water oxidation H_2_O_2_. Also, the hydrophobicity is responsible for the H_2_O_2_ production and decomposition process kinetically. Therefore, after the addition of hydrophobic polytetrafluoroethylene on BiVO_4_ surface, it achieves an average Faradaic efficiency (FE) of 81.6% in a wide applied bias region (0.6–2.1 V vs RHE) with the best FE of 85%, which is 4‐time higher than BiVO_4_ photoanode. The accumulated H_2_O_2_ concentration can reach 150 µm at 1.23 V versus RHE under AM 1.5 illumination in 2 h. This concept of modifying the catalyst surface microenvironment via stable polymers provides a new approach to tune the multiple‐electrons competitive reactions in aqueous solution.

## Graphical abstract

The PTFE is coated on the BiVO_4_ photoanode to build a hydrophobic microenvironment. It can not only favor the 2‐electron H_2_O_2_ pathway in thermodynamics via the confined O_2_ gas on polymer surface, but also promote the H_2_O_2_ desorption in kinetics by the hydrophobicity. This work opens up new possibilities of utilizing surface microenvironment to tune the liquid multiple‐electrons competitive reactions.

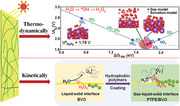

## 1 Introduction

Photoelectrochemical (PEC) water splitting to H_2_ and O_2_ has been extensively investigated over five decades since the great discovery by Fujishima and Honda in 1972.^[^ ^1^ ^]^ However, the reduction reaction efficiency of H_2_O to H_2_ is limited by the oxidation reaction of H_2_O to O_2_ due to its sluggish kinetic process with four steps of proton‐coupled electron transfer.^[^ ^2^ ^]^ Therefore, it's imperative to explore some alternative oxidative reactions, which not only efficiently match the reduction reaction, but also the production is value‐added. PEC water oxidative H_2_O_2_ production substituting the O_2_ evolution reaction (OER), by coupling with cathodic H_2_ generation of solar water splitting, has been gradually considered as a promising approach because of: i) faster kinetic process of 2‐electron transfer than OER of 4‐electron transfer; ii) more easily separation of gas–liquid products; iii) higher economic value H_2_O_2_ products.^[^ ^3^ ^]^ However, PEC water oxidative H_2_O_2_ reaction also faces great challenges, which mainly need to overcome a thermodynamically favorable OER and H_2_O_2_ decomposition reaction, as shown in Equations (1)–(3).^[^ ^3^, ^4^ ^]^

(1) $$2H_{2}O+4e^{-}/h^{+}\to \;O_{2}+2H_{2}\;\left(E^{0}=+1.23\;V\;\mathrm{vs}\;\mathrm{RHE}\right)$$

(2) $$2H_{2}O+2e^{-}/h^{+}\to H_{2}O_{2}+H_{2}\;\left(E^{0}=+1.77\;\;V\;\mathrm{vs}\;\mathrm{RHE}\right)$$

(3) $$H_{2}O_{2}\to O_{2}+2H^{+}+2e^{-}\;\left(E^{0}=+0.68\;V\;\mathrm{vs}\;\mathrm{RHE}\right)$$

The 2‐electron water oxidation reaction (WOR) for H_2_O_2_ production has been examined over many metal oxides, such as ZnO,^[^ ^5^ ^]^ Bi_2_WO_6_,^[^ ^6^ ^]^ TiO_2_,^[^ ^7^ ^]^ BiVO_4_,^[^ ^7^, ^8^ ^]^ etc. Among these oxides, BiVO_4_ is the most promising photoanode for 2‐electron WOR due to its suitable bandgap for light‐harvesting, deep valance band edge, and favorable thermodynamic activity trend for H_2_O_2_ evolution. For this reaction, many strategies including surface passivation,^[^ ^9^ ^]^ heterojunction construction,^[^ ^10^ ^]^ heteroatom doping,^[^ ^8^, ^11^ ^]^ crystal surface regulation,^[^ ^12^ ^]^ etc. have been devoted to develop the highly active BiVO_4_‐based photoanode. Such strategies mainly focus on tailoring the BiVO_4_ surface electronic property, while the PEC water splitting reaction is still carried out at an inherent hydrophilic solid–liquid interface, which will cause the in situ produced H_2_O_2_ to be decomposed before desorption in long‐term operation.^[^ ^3^, ^4^ ^]^ Therefore, the hydrophobic gas‐liquid‐solid three‐phase interface is desired to enhance the kinetic of H_2_O_2_ desorption to make the H_2_O_2_ separation before the reaction reaches equilibrium. In this case, creating an appropriate hydrophobic electrode surface is a promising strategy to kinetically regulate the O_2_/H_2_O_2_ production formation rate, thereby enhancing the H_2_O_2_ selectivity and accumulation amount.

The hydrophobicity of electrode surface was previously tuned through polymer modification.^[^ ^13^ ^]^ Other than that, the polymer can also accumulate the gas molecules, which can further interact with the catalysts’ surface and thus effectively regulate the intermediate binding energy strength for completely different reaction paths.^[^ ^14^ ^]^ Recently, Xia et al.^[^ ^14^ ^]^ demonstrated that the selectivity of water oxidation could be altered from 4‐electron WOR for O_2_ product to 2‐electron WOR for H_2_O_2_ evolution by coating the electrocatalysts (C, Ni) with a hydrophobic polymer polytetrafluoroethylene (PTFE). It was suggested that the excellent H_2_O_2_ selectivity attributed to the shift of the *OH intermediate binding energy through a less oxidized catalyst surface caused by the locally confined O_2_ gas on PTFE surface. This catalytic concept can also be applied in photo‐electrochemical field. Combined with the BiVO_4_ catalyst, the confined O_2_ gas molecules on polymer surface have no effect on its oxidation degree, while the possibility of their effect on the reaction intermediate species for tuning its binding energy strength need to be done to experimentally prove and support these hypotheses.

Herein, we present a hydrophobic gas‐liquid‐solid three‐phase interface to build a tailored catalytic microenvironment to regulate the PEC water oxidation reaction pathway in thermodynamics and kinetics. We show that the hydrophobic polymer can not only in‐situ confine O_2_ gas close to active sites to shift the *OH intermediate thermodynamically for H_2_O_2_ evolution, but also help to tune the release rate of the gas/liquid product dynamically for H_2_O_2_ generation and accumulation. Using BiVO_4_ as the model system, it is found that O_2_ evolution from 4‐electron transfer and H_2_O_2_ decomposition can be significantly inhibited after the PTFE coating, achieving 4‐times higher H_2_O_2_ Faraday efficiency (FE) of 85% in a widely applied bias range (0.6–2.1 V vs RHE). The PEC water oxidative H_2_O_2_ performance is further investigated by in situ Raman spectra, which intuitively prove that the *OH intermediates play a decisive role in promoting the PEC water oxidative H_2_O_2_ activity.

## 2 Results and Discussion

**Figure 1:**
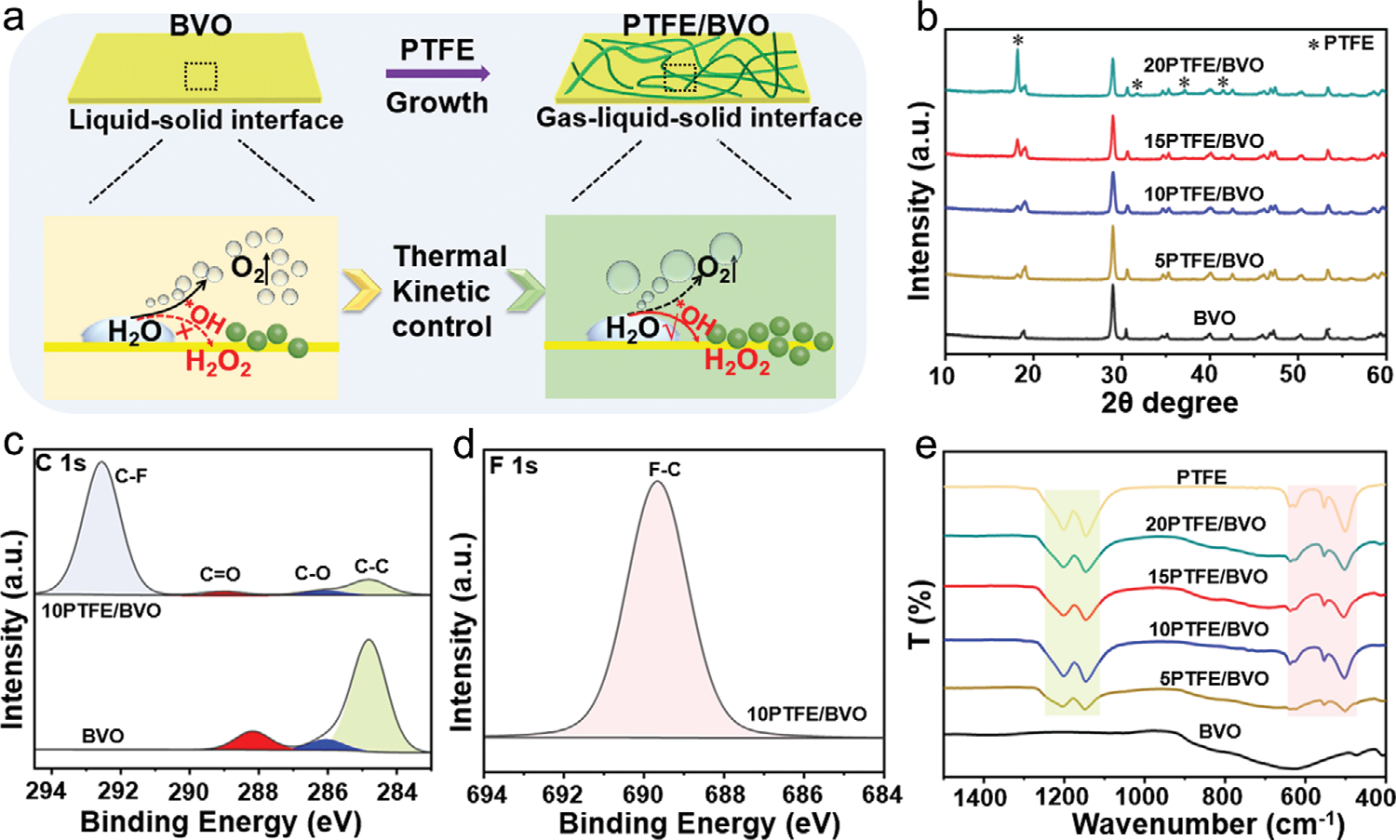
a) Schematic illustrations for regulating the ratios of O_2_ gas and H_2_O_2_ liquid product of BVO and PTFE/BVO photoanodes. b) XRD patterns of BVO and PTFE/BVO composite photoanodes. High‐resolution XPS spectra of BVO and 10PTFE/BVO photoanodes: c) C 1s and d) F 1s. e) FT‐IR spectra of BVO and PTFE/BVO photoanodes.

The synthesis route of the aerophilic–hydrophobic PTFE/BVO photoanode is displayed in Figure S1, Supporting Information, where the BiVO_4_ electrode was prepared by electrodeposition based on a previous report.^[^ ^15^ ^]^The PTFE, as one of the most hydrophobic materials,^[^ ^16^ ^]^ is chosen as the overlayer framework for allowing the assemblage of the gaseous product O_2_ of water on the catalyst surface in aqueous photoelectrocatalysis. The confined O_2_ helps to influence the reaction intermediates to thermodynamically regulate the reaction path of PEC water oxidation. Also the hydrophobicity favors to tune the adsorption or desorption capacity of the gaseous O_2_ and liquid H_2_O_2_ product in water oxidation reaction (**Figure** **1**a). In the work, we investigated the BiVO_4_ coated by PTFE with different content toward PEC H_2_O oxidative H_2_O_2_ evolution. In addition, we also prepared other hydrophobic material coated on BiVO_4_ photoanode to further confirm the catalytic microenvironmental regulation strategy.

The successful loading of PTFE on BiVO_4_ surface is confirmed by a series of systematic characterizations. As presented by XRD in Figure 1b, the peaks with “*” correspond to the PTFE diffraction peaks in PTFE/BVO photoanodes, and the other peaks are indexed to the monoclinic phase BiVO_4_.^[^ ^17^ ^]^ The PTFE diffraction peaks are displayed in Figure S2, Supporting Information. The XPS spectra in Figure 1c,d display an additional peak pertaining to —CF*_x_* deriving from the PFTE located at 292.5 eV in the C1s and 689.7 eV in the F 1s of the PTFE‐coated sample.^[^ ^18^ ^]^ A significant shift to higher binding energies for Bi 4f, V 2p, and O 1s XPS spectra peaks after coating with PTFE indicates a decrease in electron density in metal sites of the bulk phase (Figure S3, Supporting Information), which can be ascribe to the electron‐withdrawing character of PTFE. Additionally, the FT‐IR measurements in Figure 1e show that two significant peaks appear at 500–650 and 1150–1250 cm^−1^ ascribing to —CF_2_ and —CF_3_ functional group, further indicating the successful coating of PTFE.^[^ ^16^, ^19^ ^]^ Impressively, no peak shifts of BiVO_4_ and PTFE/BVO composite samples are observed in the XRD, FT‐IR, and Raman (Figure S4, Supporting Information), which confirms the unchanged monoclinic BiVO_4_ after the physical adsorption of PTFE.

**Figure 2:**
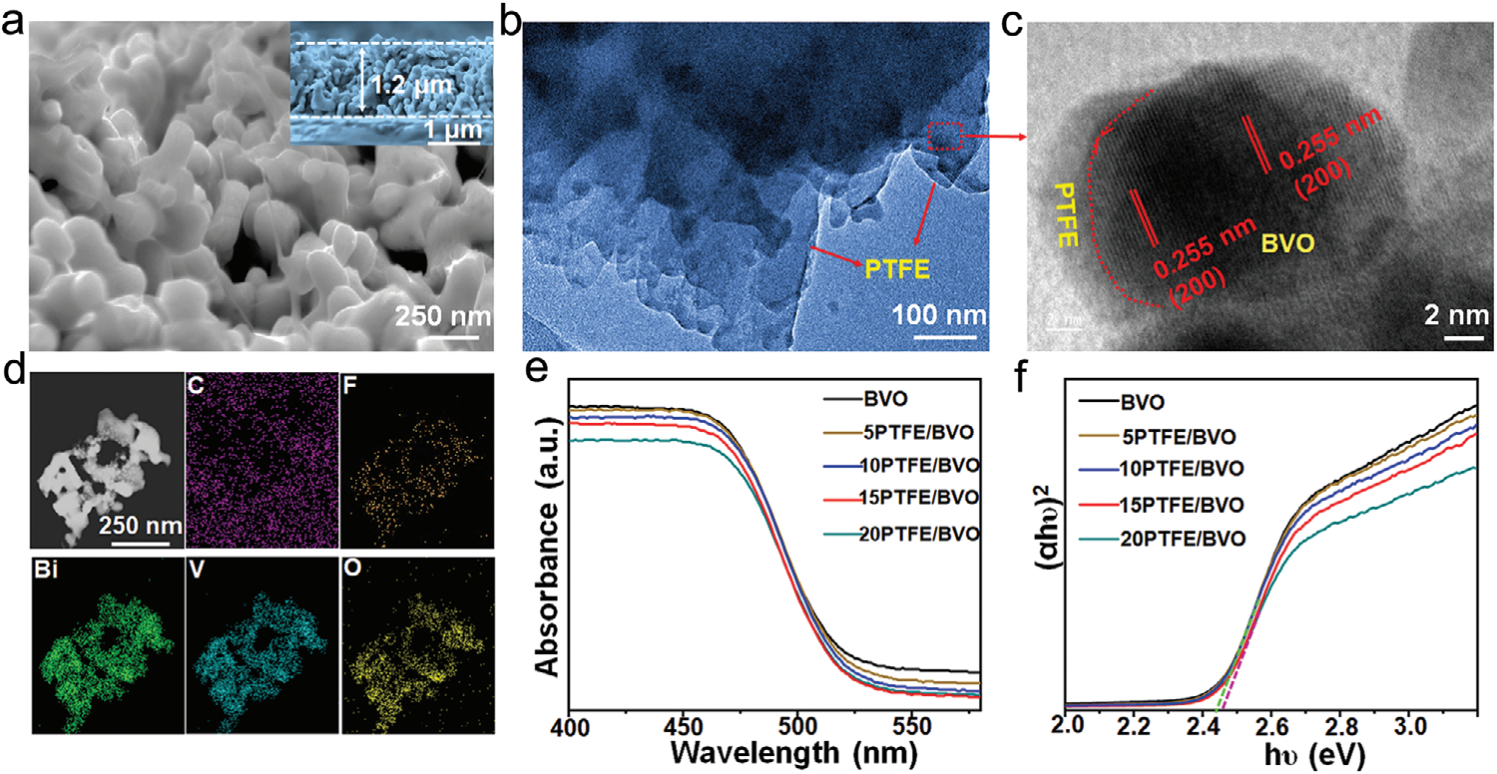
a) Top‐view and cross‐sectional SEM images of 10PTFE/BVO photoanode. b,c) HR‐TEM and d) STEM‐EDX elemental mapping images of 10PTFE/BVO photoanode. e) UV–vis absorption spectra of BVO and PTFE/BVO composite photoanodes. f) Plots of (*α*h*υ*)^2^ versus photo‐energy (h*υ*) for BVO and PTFE/BVO composite samples.

The top (**Figure** **2**a) and cross‐section (inset in Figure 2a) views of microstructures of the 10PTFE/BVO electrode demonstrate the similar morphology to pure BiVO_4_ (Figure S5, Supporting Information) because of a very thin filiform PTFE film layer on the BiVO_4_ surface. With the increase of PTFE content in PTFE/BVO composites, the filaments can be seen obviously (Figure S5, Supporting Information). The high‐resolution transmission electron microscopy (HR‐TEM) images of 10PTFE/BVO electrode in Figure 2b,c show that the BiVO_4_ nanoparticles are covered by an obvious PTFE layer. The lattice spacing of 0.255 nm corresponds to the (2 0 0) planes of monocline BiVO_4_. The element mapping of the 10PTFE/BVO electrode clearly show that Bi, O, V, and F are uniformly distributed in the entire area (Figure 2d). The UV–vis absorption spectra of PTFE/BiVO_4_ photoanodes exhibit a blue shift compared to pristine BiVO_4_ (Figure 2e), corresponding to the band gap varies from 2.43 to 2.45 eV (Figure 2f), illustrating that the change in electron structure of BiVO_4_ caused by PTFE polymer has little effect on its band gap. In addition, the aerophilic heterogeneous interface affecting the adsorption and desorption ability of the gas‐liquid products should also be considered.^[^ ^20^ ^]^ With the addition of PTFE, the PTFE/BiVO_4_ samples have good hydrophobicity, in which the water angle varies from 31.0° to 122.9°, as shown in Figure S6, Supporting Information. Visually, the O_2_ gas in aqueous solution can be certified by the locally confined O_2_ bubbles for 10PTFE/BVO (Movie S1, Supporting Information) and the rapid release for pristine BiVO_4_ in the PEC process (Movie S2, Supporting Information). The concentration of the O_2_ bubbles on the photoelectric‐catalyst surface can be clearly seen in Figure S7, Supporting Information. The surface hydrophobicity would have a significant role in H_2_O_2_ release kinetics, which is expected to tune the liquid‐H_2_O_2_ and gas‐O_2_ product ratios in PEC water oxidation reaction. Significantly, proper free energy of the *OH is generally desired for thermodynamically favorable H_2_O_2_ evolution in the water oxidative H_2_O_2_ reaction process: too strong an OH binding would further oxidize *OH to *OOH, completing the 4‐electron WOR process for O_2_; too weak a binding would be favorable to release OH into the solution as hydroxyl radical product (·OH).^[^ ^4^, ^14^ ^]^ For PTFE/BVO system, the confined O_2_ molecules with the potential to donate electrons might have a significant regulatory effect on the nearby *OH and then control the *OH binding energy to a suitable position, which will be justified by the following experimental and computational part.

**Figure 3:**
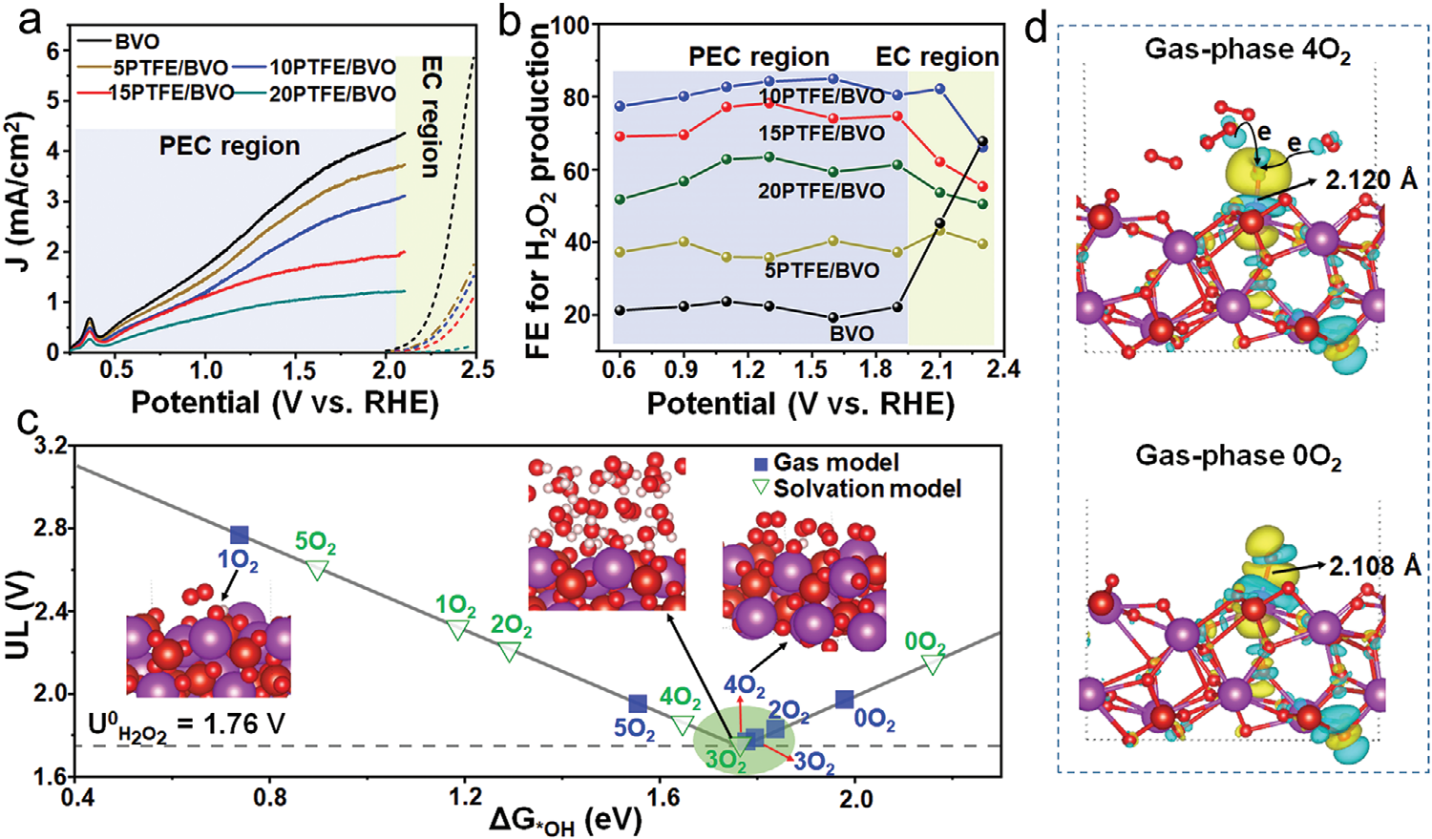
a) *J–V* curves of BVO and PTFE/BVO composite photoanodes in 1 m NaHCO_3_ electrolyte under dark (dash line) or AM 1.5 illumination (solid line). b) The calculated FEs of the PEC H_2_O_2_ evolution of BVO and PTFE/BVO composite photoanodes at various applied biases. c) 2e^−^‐WOR volcano plot as a function of the *OH binding energy (ΔG_*OH_). d) Charge redistribution in BiVO_4_(111) adsorbed by one *OH intermediate in the presence or absence of gas‐phase O_2_ (yellow, gaining electron; blue, losing electron). Atom: little red, O; big red, V; purple, Bi; white, H. The isosurface is set to 0.002 e Å^−3^.

The PEC H_2_O_2_ generation was investigated in 1 m NaHCO_3_ electrolyte with a pH value of 8.3 under AM 1.5 illumination (100 mW cm^−2^), and the H_2_O_2_ product was quantified by using *N*,*N*‐diethyl‐1,4‐phenylene‐diamine (DPD) method. As shown in **Figure** **3**a, the overall current density gradually decreases with the increasing PTFE coated, since the dielectric PTFE repels water and results in a lower electrochemical surface area (Figure S8, Supporting Information). An oxidation peak at 0.25–0.5 V versus RHE belongs to the reoxidation of V species in BiVO_4_ and the details are given in Figure S9, Supporting Information. However, the PTFE overlayer plays a positive role for the PEC H_2_O_2_ selectivity after the illumination. The corresponding real‐time Faraday efficiencies (FEs) for water oxidative H_2_O_2_ production under applied bias ranging from 0.6 to 2.1 V versus RHE are investigated in Figure 3b. In the PEC reaction process, the real‐time FEs of BiVO_4_ and PTFE/BVO photoanodes maintain at a steady stage and the PTFE overlayers indeed dramatically increase the FEs of BiVO_4_. The average FEs of H_2_O_2_ production for pristine BiVO_4_ was 21.8%, but increased to 37.8%, 81.6%, 73.8%, and 59.2% with the increasing PTFE content, respectively. The 10PTFE/BVO photoanode exhibits a maximal 4‐fold improvement in H_2_O_2_ selectivity with the best FE of 85% compared to pristine BiVO_4_. In addition, with the applied bias voltage increased, the enhanced real‐time FEs of PTFE overlayer suddenly drop and the performance of the PTFE hydrophobic layer to enhance the FEs of the BiVO_4_ photoanode disappears, which could be ascribed to the lower dark current density where the EC reactions work. The results elucidate that the PTFE coating on the BiVO_4_ surface acts a crucial role to increase the water oxidative H_2_O_2_ selectivity.

The changes in the free energy of the *OH intermediate (ΔG_*OH_) on PTFE/BVO catalysts can be predicted by the plausible effect of the O_2_. A suitable ΔG_*OH_ indicates a shift from the 4‐electrons route to 2‐electrons pathway.^[^ ^21^ ^]^ To unveil the underlying mechanism, the volcano plot for 2‐electron WOR as a function of the ΔG_*OH_ over BiVO_4_ covered with O_2_ bubbles is shown in Figure 3c, and these thermodynamic models are also given in Figures S10, S11, Supporting Information. It is found that the number of O_2_ play an important role on the *OH binding, which shift the active sites towards the activity peak of the volcano plot. When the Bi sites are surrounded by 4 O_2_, a steady state with the most appropriate ΔG_*OH_ and the highest H_2_O_2_ activity can be obtained. To simulating a real reaction environment, an explicit approach in aqueous solution is also conducted (Figure 3c). We found that the *OH binding is also affected by O_2_ molecules, further verifying that an appropriate amount of O_2_ molecules is imperative to regulate the adsorption of *OH intermediate on the BiVO_4_ catalyst and then produce a higher H_2_O_2_ in the real H_2_O system. Simultaneously, the charge redistribution of BiVO_4_ adsorbed by one OH intermediate caused by O_2_ molecules can be analyzed through the charge density difference in Figure 3d. In the presence of adequate O_2_, the Bi—O bond length between *OH and Bi site is 2.120 Å, slightly larger than that without O_2_ (2.108 Å). The weakened Bi‐O ionic bond is ascribed to the electrons transfer from the nearby O_2_ molecules to *OH, then induce the ΔG_*OH_ to adjust to a more suitable position (at the bottom of the volcano diagram). Although the thermodynamic analysis can only be taken as qualitative, it is a first step toward understanding the trend of the selectivity changes on the modified photoelectrocatalyst by locally confined O_2_ molecules. The local O_2_ confinement approach can be extended to other hydrophobic polymers. After silane modification (PDMS/BVO), the H_2_O_2_ selectivity of BiVO_4_ photoanode is also greatly promoted, confirming that the confined O_2_ plays a significant role in enhancing H_2_O_2_ selectivity (Figure S12, Supporting Information). Other thermodynamic factors involving band structure and band bending, determined by valence band (VB) XPS spectra and Mott‐Schottky plots, are also investigated (Figure S13, Supporting Information). The similar VB edges and flat‐band potentials of BiVO_4_ and 10PTFE/BVO suggest that the two factors controlling the thermodynamic products can be excluded. Moreover, the interface charge transport and separation efficiencies of BiVO_4_ and 10PTFE/BVO do not show a big difference, which is obtained by the photocurrent density measured in hole scavenger (Figure S14, Supporting Information)

**Figure 4:**
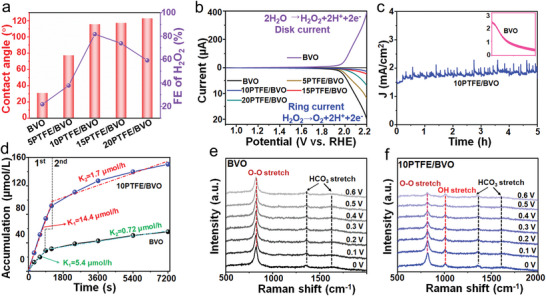
a) The real‐time FE of water oxidative H_2_O_2_ production at 1.23 V versus RHE as a function of different photoanodes surface hydrophily‐hydrophobicity. b) Rotating disk‐ring currents recorded at 1600 rpm in 1 m NaHCO_3_ electrolyte. c) *J–t* curves and d) H_2_O_2_ accumulation concentrations of BVO and 10PTFE/BVO photoanodes at 1.23 V versus RHE in 1 m NaHCO_3_ electrolyte under AM 1.5 illumination. In situ Raman spectra of e) pristine BVO and f) 10PTFE/BVO photoanodes under different applied biases in 1 m NaHCO_3_ electrolyte. The 0 V means open circuit voltage.

The PEC water oxidation products performance is not only determined by the thermodynamic effects, but also can be associated with the kinetics‐controlled processes of electron transfer. As such, the kinetics‐controlled processes involve the two competing reaction, 4‐electron WOR of O_2_ production and stepwise 2‐electron (H_2_O_2_ formation)/2‐electron transfer (H_2_O_2_ decomposition). The surface hydrophobicity might be the reason behind the kinetics‐controlled processes for selective H_2_O_2_ production. The samples with hydrophobic property (*θ* > 90°) hold higher FEs of water oxidative H_2_O_2_ production compared with the wettability (*θ* < 90°), which can be obviously seen from the plots of the FEs of water oxidative H_2_O_2_ production at 1.23 V versus RHE versus different surface hydrophily–hydrophobicity in **Figure** **4**a. It suggests that the surface hydrophobicity, especially an appropriate photoanode hydrophobic surface, is conductive to the generation of H_2_O_2_. The local hydrophobic‐aerophilic environment makes the release of O_2_ bubbles slowly and promotes the H_2_O_2_ leaving from the PTFE/BVO surface before the competitive reactions reach equilibrium, thereby kinetically increasing H_2_O_2_ production. The kinetics processes is preliminarily investigated by the ring‐disk electrode technology, in which the reaction on the disk and ring electrode is H_2_O_2_ production by BiVO_4_ and H_2_O_2_ decomposition by BiVO_4_ and PTFE/BVO, respectively (Figure S15, Supporting Information). Here, the applied bias for the ring electrode is fixed at 0.7 V versus RHE, at which the only possible EC reaction is H_2_O_2_ decomposition and no other side reactions occur. Figure 4b shows the *J–V* curves of BiVO_4_ and PTFE/BVO electrodes recorded at a scan rate of 10 mV s^−1^ in 1 m NaHCO_3_ electrolyte solution at ambient temperature at 1600 rpm. Under the same disk current generated by water oxidative H_2_O_2_ production, the ring currents come from H_2_O_2_ oxidation can reflect the effect of surface hydrophobicity.^[^ ^22^ ^]^ Remarkably, a large disk current for the water oxidative H_2_O_2_ evolution is observed, while a sharp drop ring current for the oxidative H_2_O_2_ decomposition in PTFE/BVO system. The negligible H_2_O_2_ decomposition on PTFE/BVO photoanode indicates that the hydrophobicity surface reduces the contact possibility between H_2_O_2_ and electrode to a great extent, then reducing the reoxidation and increasing the release of H_2_O_2_. In addition, the photocatalytic H_2_O_2_ decomposition by BiVO_4_ and PTFE/BVO photoanodes is also conducted in the HCO_3_^−^‐containing solution under AM 1.5 illumination. As shown in Figure S16, Supporting Information, the H_2_O_2_ concentration decreases rapidly and is almost undetectable within 60 min for the illuminated BiVO_4_ photoanode, while the retention rate of H_2_O_2_ is 85% for PTFE/BVO photoanodes during irradiant 60 min, which is basically equivalent to the optical H_2_O_2_ decomposition. Overall, the specific microenvironment indeed promotes the water oxidative H_2_O_2_ production, in which right amounts of confined O_2_ on BiVO_4_ photoanode induced by PTFE overlayer thermodynamically regulate the only intermediate *OH and maintain the thermodynamic favorable water oxidative H_2_O_2_ process, meanwhile the surface hydrophobicity of photoelectrode kinetically facilitates the H_2_O_2_ desorption in aqueous solution.

When the surface accumulated holes are located on BiVO_4_ surface, the photocorrosion of BiVO_4_ is about to initiate, then these ions (V^5+^, Bi^3+^, O^2−^) may destabilize the BiVO_4_ lattice and increase the solubility of BiVO_4_ at the surface. Since V^5+^ is more soluble than Bi^3+^ in alkaline electrolyte, the dissolution of V^5+^ will be dominant.^[^ ^23^ ^]^ The PEC water oxidative H_2_O_2_ stability over BiVO_4_ and 10PTFE/BVO photoanodes were conducted at 1.23 V versus RHE in 1 m NaHCO_3_ electrolyte, as shown in Figure 4c, from which it can be seen that the 10PTFE/BVO photoanode maintains a relatively stable J‐t curve in comparison with pristine BiVO_4_ for H_2_O_2_ production after 5 h illumination. It suggests that the PTFE protection surface can slow down the dissolution of V^5+^ of BiVO_4_, and then weaken its photocorrosion due to the surface holes accumulation.^[^ ^12^ ^]^ The above further verifies that the faster kinetics of 2‐electron WOR in 10PTFE/BVO photoanode can reduce surface holes accumulation, thereby suppressing the dissolution of V^5+^. However, the accumulation of H_2_O_2_ produced by both BiVO_4_ and 10PTFE/BVO photoanode displays a nonlinear increase as the reaction time lengthens, indicating not a first‐order dynamic process governed by electron‐hole transfer. As shown in Figure 4d, the H_2_O_2_ accumulation can be divided into two stages, in which the first stage increases rapidly, followed by a gradual decreases in the second stage. Since the H_2_O_2_ accumulated on the surface is easily re‐oxidized by photoinduced holes, which will be competed with the produced H_2_O_2_, and finally reaching dynamic equilibrium. Therefore, the two H_2_O_2_ accumulation process can be divided into 2‐electron/4‐electron WOR competing reaction and stepwise 2‐electron/2‐electron transfer of H_2_O_2_ decomposition. In the first stage, the H_2_O_2_ generation rate for 10PTFE/BVO can reach 14.4 µmol h^−1^ with an accumulation amount of 92 µmol L^−1^, while the H_2_O_2_ evolution rate for BiVO_4_ is only 5.4 µmol h^−1^ with an accumulation amount of 38 µmol L^−1^. During the second stage, despite the 10PTFE/BVO holds a slow H_2_O_2_ evolution rate of 1.7 µmol h^−1^ accompany with H_2_O_2_ decomposition, the H_2_O_2_ accumulation amount continues increase, reaching 150 µmol L^−1^ after 2 h of illumination. The above results are fully illustrated that the PTFE layer can efficiently promote the H_2_O_2_ generation and inhibit the decomposition of H_2_O_2_. Finally, the stable photocurrent density and constant FE produce a higher average solar to H_2_O_2_ efficiency of 2.02% for 10PTFE/BVO compared to 0.23% for pure BiVO_4_. Meanwhile, the obtained H_2_ amounts in the PEC water splitting over 10PTFE/BVO photoanode quantities as a function of the theoretical electron number calculated based on its photocurrent density (Figure S17, Supporting Information), confirming that H_2_ production rate is not affected by the H_2_O_2_ decomposition. Compared to previously reported strategies, such as passivation,^[^ ^3b^ ^]^ heterojunction,^[^ ^3c^ ^]^ doping,^[^ ^3d^ ^]^ crystal facets,^[^ ^4a^ ^]^ tailoring the catalyst microenvironment is a more efficient method to tune the H_2_O_2_ selectivity and accumulation, which are summarized in Table S1, Supporting Information. However, more work to prolong the H_2_O_2_ accumulation stability and improve the PEC efficiency of BiVO_4_ photoanode will be the topic of subsequent research for future possible application. The XRD, SEM, TEM, and XPS results of 10PTFE/BVO photoanode after the long‐term H_2_O_2_ evolution are shown in Figures S18–S21, Supporting Information. It can be observed that there are no detectable changes in the crystal structure, morphology, and component, confirming a good structure stability for PEC H_2_O_2_ evolution.

The specific reaction pathway for the possible water oxidation reaction (4‐electron WOR, 2‐electron WOR) are as follows:^[^ ^4^, ^24^ ^]^

(4) $$\mathrm{The}\;4-\mathrm{electron}\;\mathrm{WOR}\;(2H_{2}O\to O_{2}+4H^{+}+4e^{-})$$

(4a) $$*+H_{2}O\to *\mathrm{OH}+H^{+}+e^{-}$$

(4b) $$*\mathrm{OH}\to *O+H^{+}+e^{-}$$

(4c) $$*O+H_{2}O\to *\mathrm{OOH}+H^{+}+e^{-}$$

(4d) $$*\mathrm{OOH}\to *+O_{2}+H^{+}+e^{-}$$

(5) $$\mathrm{The}\;2-\mathrm{electron}\;\mathrm{WOR}\;(2H_{2}O\to H_{2}O_{2}+2H^{+}+2e^{-})$$

(5a) $$*+H_{2}O\to *\mathrm{OH}+H^{+}+e^{-}$$

(5b) $$*\mathrm{OH}+H_{2}O\to H_{2}O_{2}+H^{+}+e^{-}$$

In order to explore the process of producing H_2_O_2_ by PEC water oxidation via BiVO_4_ photoanode with PTFE hydrophobic layer in 1 m NaHCO_3_ electrolyte, in situ Raman spectra were investigated. The electrochemical cell is based on a homemade square teflon disk, in which the opposite electrode and the reference electrode are slender copper foil and Ag/AgCl, respectively. The test was performed at 0–0.6 V versus RHE potentials for 10 min per potential. As shown in Figure 4e,f and Figure S22, Supporting Information, two characteristic Raman peaks at 1336 and 1605 cm^−1^ attributing to the stretching of HCO_3_^−[^ ^8a^ ^]^ can be observed in both of the synthesized BiVO_4_ and PTFE/BVO photoanodes. In the Raman spectra of the BiVO_4_ photoanode, a strong signal at 830 cm^−1^ deduced as the O—O stretching bond^[^ ^25^ ^]^ appears, indicating the 4‐electron WOR dominates. However, the O—O bond in the PTFE/BVO photoanodes gradually decrease with the increasing applied bias voltage, which suggests that the 4‐electron WOR path for O_2_ generation is effectively suppressed after the PTFE hydrophobic layer loaded. Meanwhile, a new peak at 1020 cm^−1^ associating with either OH or superoxide species (·OH)^[^ ^26^ ^]^ in PTFE/BVO photoanode rises. The EPR spectrum used to characterize ·OH free radicals^[^ ^27^ ^]^ shows no signal in the 10PTFE/BVO sample (Figure S23, Supporting Information). The above results indicate that loading the PTFE hydrophobic layer can effectively inhibit the transformation of *OH to *OOH intermediates and accelerate the generation of *OH to H_2_O_2_ on BiVO_4_ surface, then selectively turn the oxidation pathway of water oxidation from 4‐electron WOR to 2‐electron WOR.

## 3 Conclusion

In this work, we have successfully confirmed that the surface microenvironmental modification of BiVO_4_ photoanode upon polymer loading can greatly enhance the PEC H_2_O_2_ selectivity and accumulation, in which the average FE of H_2_O_2_ is increased by a factor of 4 and the saturated H_2_O_2_ concentration can reach 150 µm at 1.23 V versus RHE under AM 1.5 illumination for continuous 2 h after the addition of PTFE. From experiments and DFT studies, we can explain this improvement by the two main factors: 1) the energetics of WOR of BiVO_4_ photoanode is regulated to favor the 2‐electron H_2_O_2_ pathway in thermodynamics via the confined local O_2_ gas on PFFE surface; 2) the H_2_O_2_ desorption is greatly promoted in aqueous solution and H_2_O_2_ decomposition is effectively inhibit in kinetics by the PTFE hydrophobicity. Impressively, the resultant PFFE/BVO photoanode displays better photocurrent density stability than that of BiVO_4_. This work provides a triple effect of favorable H_2_O_2_ generation, suppressed OER route and H_2_O_2_ decomposition via the design of BiVO_4_ photoanode surface microenvironment, and the approach has the potential to be applicable to other heterogenous photoelectrochemical system.

## 4 Experimental Section

### Preparation of PTFE/BVO Photoanodes

BiVO_4_ (BVO) photoanodes were prepared according to the method of Lee and Choi's method.^[^ ^15^ ^]^ The PTFE/BVO composite photoanodes were further constructed by spin‐coating approach, for which BVO was spun by the same amount of PTFE solutions with different concentrations (5 wt%, 10 wt%, 15 wt%, and 20 wt%) at 1500 rpm for 150 s. Then the above precursors were annealed at 350 °C for 30 min in air atmosphere to form PTFE/BiVO_4_ electrodes with different proportions, denoted as *x*PTFE/BVO (*x* is the concentration of PTFE).

### Preparation of PDMS/BVO Photoanodes

The BiVO_4_ electrode was soaked in 9 mL tetrahydrofuran (THF) solution containing 3 g polydimethylsiloxane (PDMS) and stirred gently for 24 h. Then the above electrode precursor was illuminated with UV‐light and the light intensity was controlled at 10 mW cm^−2^. After illuminated for 2 h, the resulting PDMS/BVO photoanode was washed with THF and deionized water and dried at room temperature.

### Characterizations

The structure and chemical properties of the as‐synthesized samples was confirmed with X‐ray diffraction (XRD, Cu K*α*, Rigaku SmartLab), field emission scanning electron microscope (SEM, Hitachi S‐8200), transmission electron microscope (TEM) and energy dispersive X‐ray spectroscopy (EDX) (JEOL JEM‐ARM 200F), Raman microscope (HORIBA Xplora Plus) using a 50× objective excited by 532 nm laser light with a power of 20 mW, UV–vis diffuse reflectance spectra (DRS, Shimadzu UV‐2600), X‐ray photoelectron spectroscopy (XPS, Al K*α*, Escalab 250Xi) and all the peaks calibrated with a C 1s spectrum at a binding energy of 284.6 V, electron paramagnetic resonance spectra (EPR, Bruker EMX‐10/12‐type spectrometer) in 1 m NaHCO_3_ electrolytes under Xe lamp irradiation with a 420 nm cutoff filter, the contact angles (JC2000D1) with a NaHCO_3_ droplet on the samples. The ring‐disk current was carried out in N_2_‐saturated 1 m NaHCO_3_ solution at 1600 rpm with a scan rate of 10 m s^−1^. The disk electrode was BiVO_4_ with a loading mass of 0.24 mg cm^−1^, and the ring electrodes were BiVO_4_, *x*PTFE/BVO (*x* = 5, 10, 15, and 20) with a loading mass of 0.1 mg cm^−1^. The electrochemical cell was based on a self‐made square Teflon dish. The counter electrode and the reference electrode were a slender Pt foil and an Ag/AgCl.

### Photoelectrochemical Measurements

The photoelectrochemical (PEC) performance was evaluated in an H‐type three‐electrode quartz electrolytic cell with Ag/AgCl as reference electrode, Pt as counter electrode, Nafion film as the ion exchange membrane, and 1 m NaHCO_3_ solution (pH = 8.3) as electrolyte using a CHI 660E electrochemical workstation at room temperature of 25 °C. The illumination source was a 300 W Xe arc lamp with an AM 1.5G filter (100 mW cm^−2^, CEL‐HXF300) and all electrodes were illuminated from the back‐side. All illuminated areas were 1.5 cm^2^. Linear sweep voltammetry (LSV) was monitored while sweeping the potential in the positive direction with a scan rate of 10 mV s^−1^. The potential versus Ag/AgCl reference electrode was converted to the potential versus RHE according to the Nernst equation: *E* (vs RHE) = *E* (vs Ag/AgCl) + 0.0591 × pH +0.197. Flat‐band potential measurements were measured using Mott–Schottky plots at potentials varying between 0 and 0.6 V with a frequency of 1 KHz.

### Production Measurements

The evolution of H_2_O_2_ was detected by *N,N*‐diethyl‐1,4‐phenylenediamine method. The solution of DPD was prepared by dissolving 0.1 g DPD in a 10 mL of 0.05 m sulfuric acid solution. The POD solution was made by dissolving 10 mg POD in 10 mL deionized water and stored in the refrigerator for use. Potassium phosphate buffer was prepared by mixing 49.85 mL deionized water, 43.85 mL of 1 m potassium dihydrogen phosphate and 6.3 mL of 1 m potassium phosphate. After the photoelectric reaction, 1 mL of the reacted solution was put into a test tube containing 0.4 mL potassium phosphate buffer, 3 mL water, 0.05 mL DPD, and 0.05 mL POD and shaken for 2 min. The obtained solution was analyzed by UV–vis spectroscopy. The Faradaic efficiencies of H_2_O_2_ were calculated as shown in Equation (4):

(6) $$\begin{array}{ccc} \eta \;\left(H_{2}O_{2}\right) & = & \frac{\mathrm{amount}\;\mathrm{of}\;\mathrm{generated}\;H_{2}O_{2}}{\mathrm{theoretical}\;\mathrm{amount}\;\mathrm{of}\;H_{2}O_{2}}\times 100\\ & = & \frac{\mathrm{amount}\;\mathrm{of}\;\mathrm{generated}\;H_{2}O_{2}}{\mathrm{amount}\;\mathrm{of}\;\mathrm{generated}\;\mathrm{electrons}/2}\times 100 \end{array}$$

The solar to H_2_O_2_ efficiency can be calculated from the *J–V* curve and FE of H_2_O_2_ evolution using the following Equation 5:

(7) $$\eta_{\mathrm{STH}}\;=\left[\frac{J_{\mathrm{SC}}\left(\mathrm{mA}cm^{-2}\right)\times E_{H_{2}O_{2}/2H_{2}O}^{1.77}\times \eta_{\mathrm{FE}}}{P_{\mathrm{total}}\left(\mathrm{mW}\;cm^{-2}\right)}\right]$$

Herein, no electrocatalysis occurs at 1.23 V versus RHE, the *J*_sc_ for 10PTFE/BVO was 1.34 mA cm^−2^, *η*_FE_ for H_2_O_2_ production was 85%, *P*_total_ was 100 mW cm^−2^, the solar to H_2_O_2_ efficiency can be calculated to 2.02%.

The H_2_ evolution was measured in an on‐line automatic closed circulation reactor system (Perfectlight Sci&Tech Co., Ltd., Labsolar‐6A) that was connected with an online gas chromatograph (GC 9790, TCD, 5 Å molecular sieve columns and Ar carrier).

### Computational method

All calculations were carried out using DFT with the Perdew–Burke–Ernzerhof (PBE) form of the generalized gradient approximation (GGA) functional.^[^ ^28^ ^]^ The Vienna ab initio simulation package (VASP) was employed.^[^ ^29^ ^]^ The energy cutoff for plane wave expansions was set to 450 eV, and the energy (converged to 1e^−5^ eV atom^−1^) and force (converged to −2e^−2^ eV Å^−1^) were set as the convergence criteria for geometry optimization. A BiVO_4_ (111) periodic slab model which contain 48 atoms (Bi_8_V_8_O_32_) was constructed to simulate the BiVO_4_ (111) surface. The Brillouin zones were sampled with the gamma‐centered Monkhorst‐Pack^[^ ^30^ ^]^ (2 × 4 × 1) k‐points meshes for BiVO_4_ (111) surface model. As for the slab model, a vacuum space of 15 Å was added to the slab model to avoid interaction between periodic images. In addition, the DFT‐D3 method^[^ ^31^ ^]^ was included to improve the description of the long‐range weak van der Waals (vdW) interaction for all DFT calculations. The free energy correction for adsorbate is calculated by VASPKIT.^[^ ^32^ ^]^ To investigate the effect of surface adsorbed *O_2_ on catalytic activity, we calculated the *O_2_ coverage‐dependent *OH binding free energies (ΔG_*OH_), which is a key intermediate in electrochemical H_2_O_2_.^[^ ^33^ ^]^ Charge redistribution was defined as Δ*ρ* = *ρ*_Slab+OH_ − *ρ*_Slab_ − *ρ*_OH_, where *ρ*_Slab+OH_, *ρ*_Slab_, and *ρ*_OH_ denote the charge distribution of the whole adsorption system, BiVO_4_ (111) surface with/without the *O_2_ coverage and *OH.

## Conflict of Interest

The authors declare no conflict of interest.

## Supporting information

Supporting Information

Supplemental Movie 1

Supplemental Movie 2

## Data Availability

The data that support the findings of this study are available from the corresponding author upon reasonable request.
