# Supplementary material for: Tailored BiVO4 Photoanode Hydrophobic Microenvironment Enables Water Oxidative H2O2 Accumulation
Source: Adv Sci (Weinh). 2023 Mar 31;10(15):2300169. doi: 10.1002/advs.202300169 (PMC10214242; doi:10.1002/advs.202300169)
Supplement: Supplementary file 1 — Supporting Information [file ADVS-10-2300169-s001.pdf]

ADVANCED SCIENCE
Open Access

Supporting Information

for *Adv. Sci.*, DOI 10.1002/advs.202300169

Tailored $BiVO_4$ Photoanode Hydrophobic Microenvironment Enables Water Oxidative H_2O_2 Accumulation

Man Ou, Mei Geng, Xiangle Fang, Wenfan Shao, Fenghong Bai, Shipeng Wan, Caichao Ye, Yuping Wu and Yuhui Chen**

Supporting Information

Tailored $BiVO_4$ photoanode hydrophobic microenvironment enables water oxidative H_2O_2 accumulation

Man Ou[‡a], *Mei Geng*[‡a], *Xiangle Fang*[a], *Wenfan Shao*[a], *Fenghong Bai*[a], *Shipeng Wan*[c], *Caichao Ye**[b], *Yuping Wu*[a], *Yuhui Chen**[a]

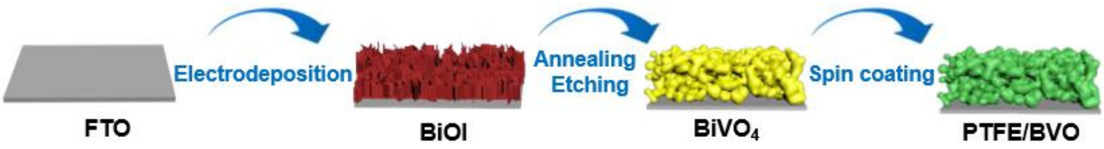

Figure S1. Schematic illustration of the synthesis procedure of $BiVO_4$ and PTFE/BVO photoanodes.

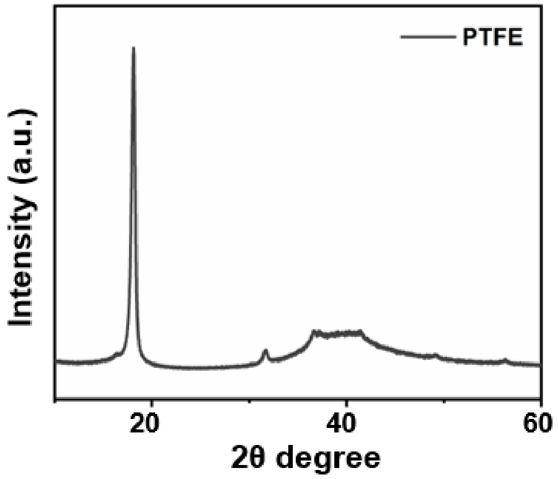

Figure S2. XRD patterns of PTFE.

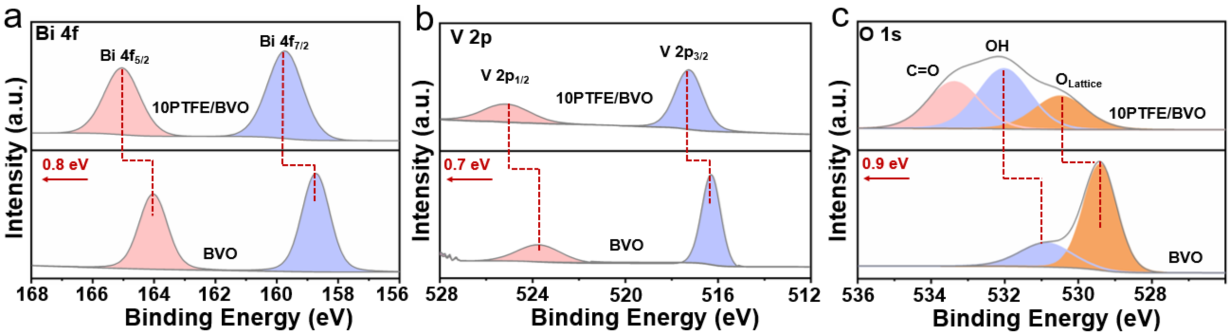

Figure S3. High resolution XPS spectrum of a) Bi 4f, b) V 2p, c) O 1s of pristine $BiVO_4$ and 10PTFE/BVO.

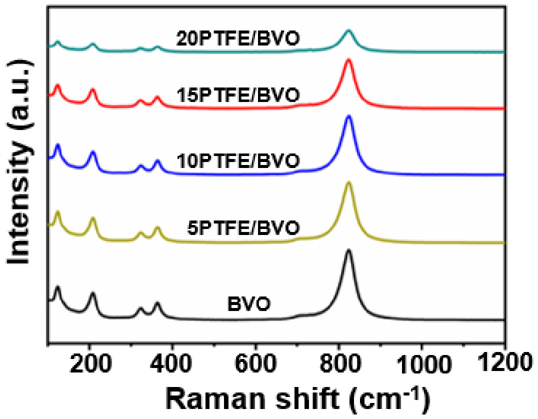

Figure S4. The Raman shifts of the synthetic $BiVO_4$ and PTFE/BVO photoanodes.

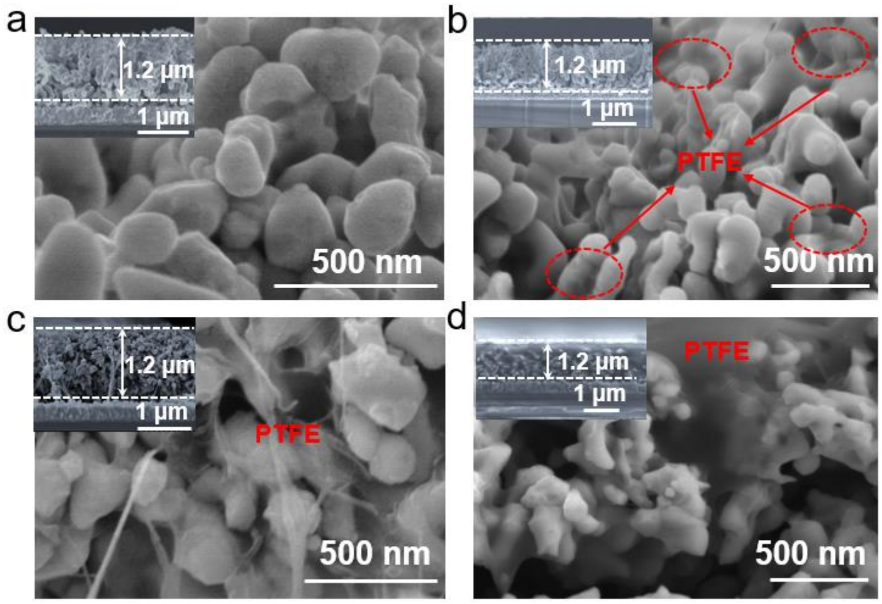

Figure S5. Top-view and cross-sectional SEM images of a) pure $BiVO_4$, b) 5PTFE/BVO, c) 15PTFE/BVO and d) 20PTFE/BVO components.

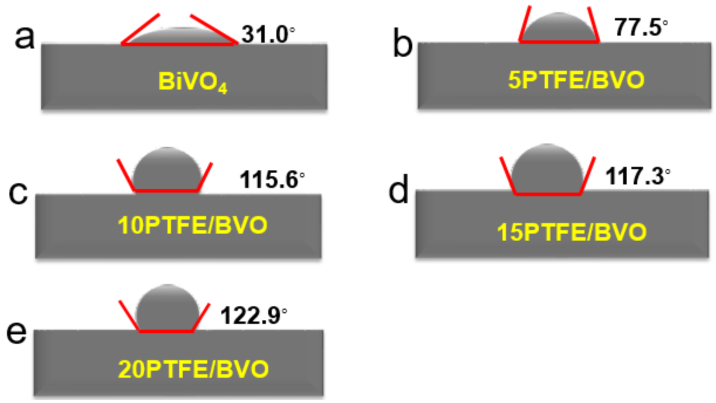

Figure S6. Contact angles of liquid H_2O on a) $BiVO_4$, b) 5PTFE/BVO, c) 10PTFE/BVO, d) 15PTFE/BVO and e) 20PTFE/BVO samples.

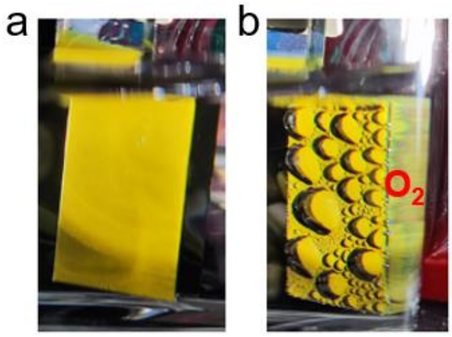

Figure S7. Digital photos for a) pure $BiVO_4$ and b) 10PTFE/BVO photoanode at 1.23 V vs. RHE in a 1 M $NaHCO_3$ electrolyte.

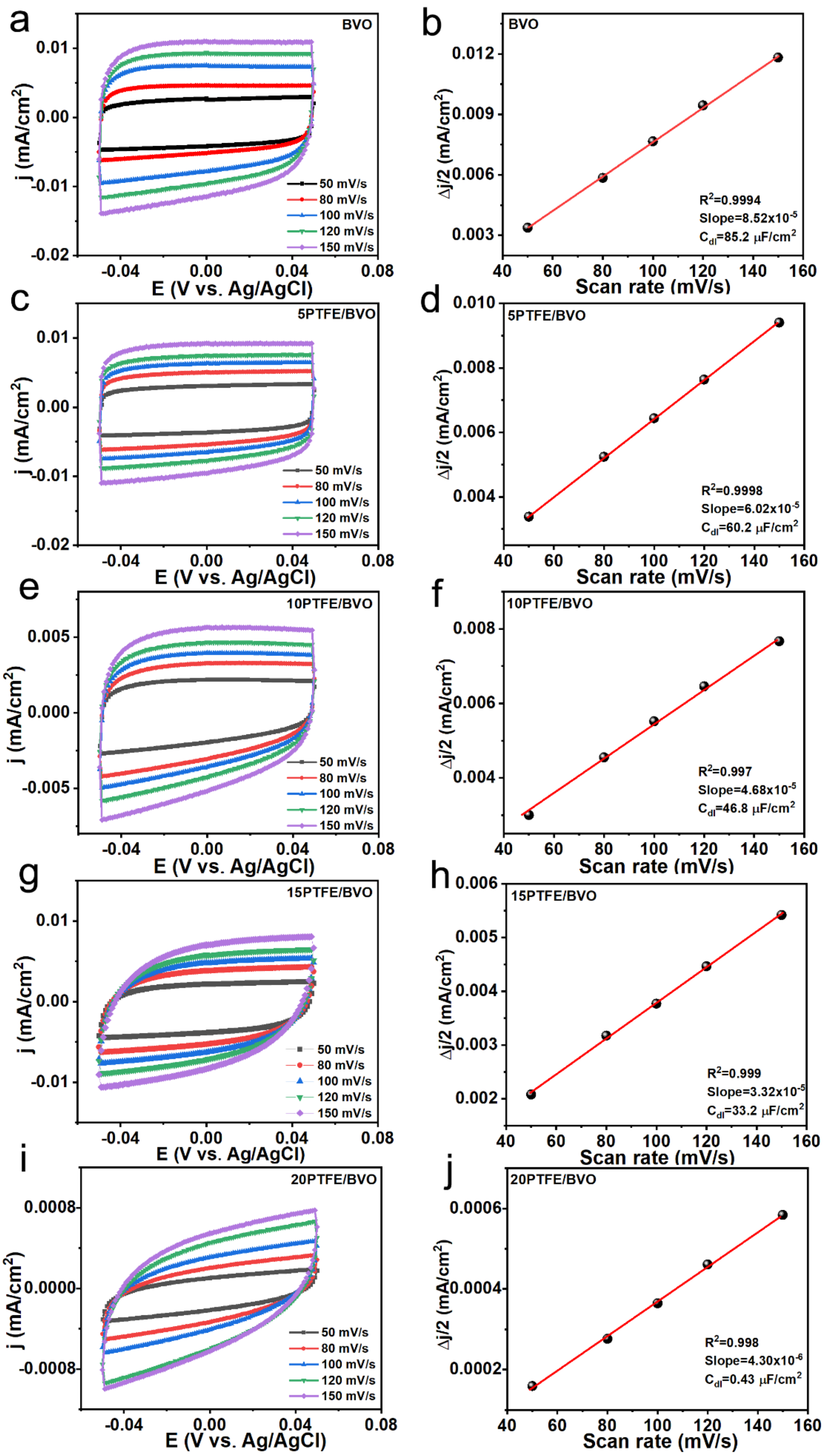

Figure S8. Double-layer capacitance measurements for pure $BiVO_4$ and PTFE/BVO composites.

The electrochemical surface area (ECSA) is derived from the electrochemical double layer capacitance (C_{dl}) of the catalytic surface, which is obtained by testing the capacitive current associated with double-layer charging from the scan-rate dependence of cyclic voltammetry. To determine the C_{dl} for various electrodes, multiple cyclic voltammetry experiments were performed with the scan rates from 50 to 150 mV/s. A linear regression was plotted between the capacitive current density differences (Δj) and the scan rate (Figure S8) in the potential window, in which the fitting slope of the line was twice the C_{dl}. Herein, the C_{dl} value is linearly promotional to the ECSA. It can be clearly seen that the C_{dl} value gradually decreases with the addition of PFTE in the $BiVO_4$-based photoanode system. Therefore, the ECSA of $BiVO_4$ decreases with the increase of PTFE surface loaded.

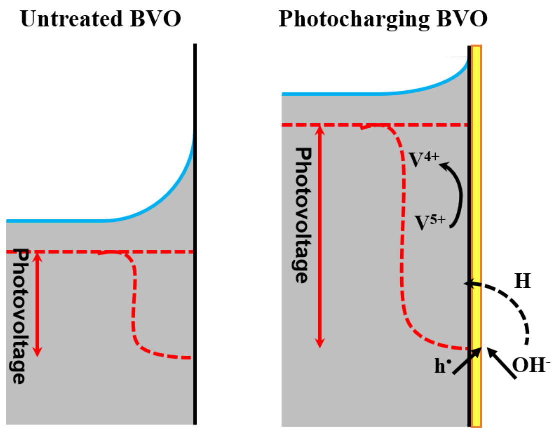

Figure S9. Band diagrams of BVO under illumination before and after the photocharging treatment and the photocharging mechanism model.

Based on the literature[1], the hydroxyl in the alkaline electrolyte is easily adsorbed on the $BiVO_4$ surface, then the hydrogen get incorporated into the $BiVO_4$ lattice via intercalation into interstitial sites after the photocharging (photoelectrochemical procedure), leading to the formation of positively charged defects. For the sake of conservation of charge, the V^{5+} in $BiVO_4$ is most likely reduced to the negatively charged V species. The specific mechanism is shown in **Figure S9**. Then, the produced reduced V species are reoxidized to V^{5+} by the surface accumulated holes as the phoelectro-catalytic oxidation reaction proceeds. Therefore, the peak in the range of 0-0.5 V is corresponding to the reoxidation of V species in $BiVO_4$. Such result has also been commonly reported by others in photoelectrochemical water oxidation systems involving $BiVO_4$[2].

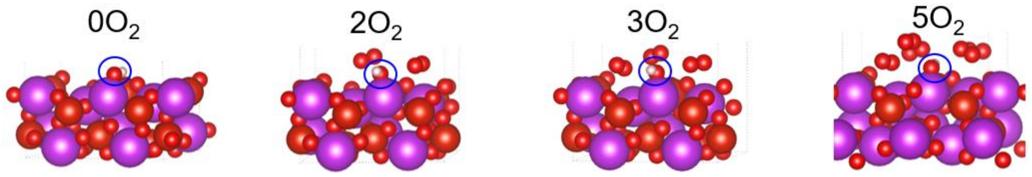

Figure S10. The calculation models of different confined O_2 on the $BiVO_4$ (111) unit cells with *OH 2e- water oxidation reaction intermediates. The adsorbed intermediates are highlighted in blue circles. Atom: little red, O; big red, V; purple, Bi; white, H.

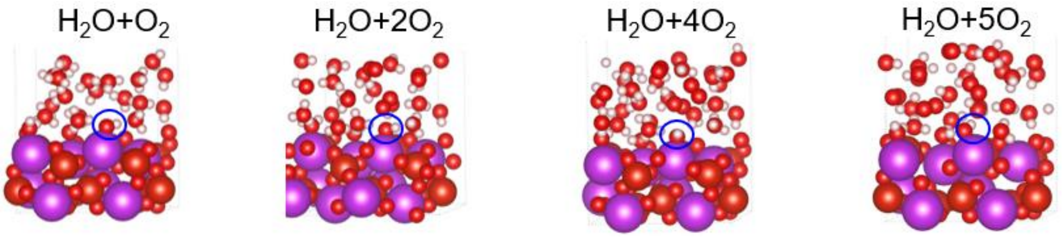

Figure S11. The model diagram of the influence of different confined O_2 to the *OH intermediates adsorbed at the Bi site in $BiVO_4$ (111) unit cells under the real H_2O circumstances. The adsorbed intermediates are highlighted in blue circles. Atom: little red, O; big red, V; purple, Bi; white, H.

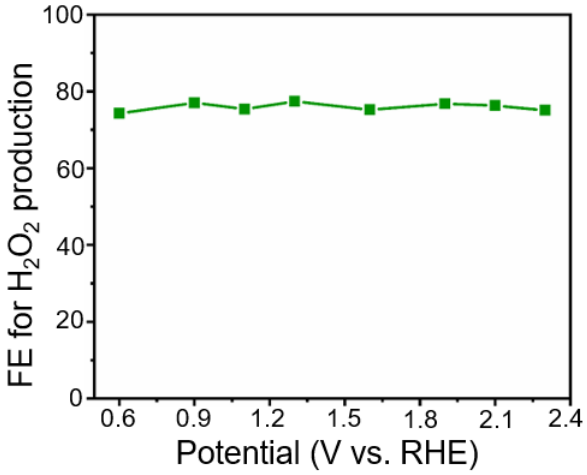

Figure S12. The calculated real-time FE of the H_2O_2 evolution of the PDMS/BVO photoanode at various applied biases.

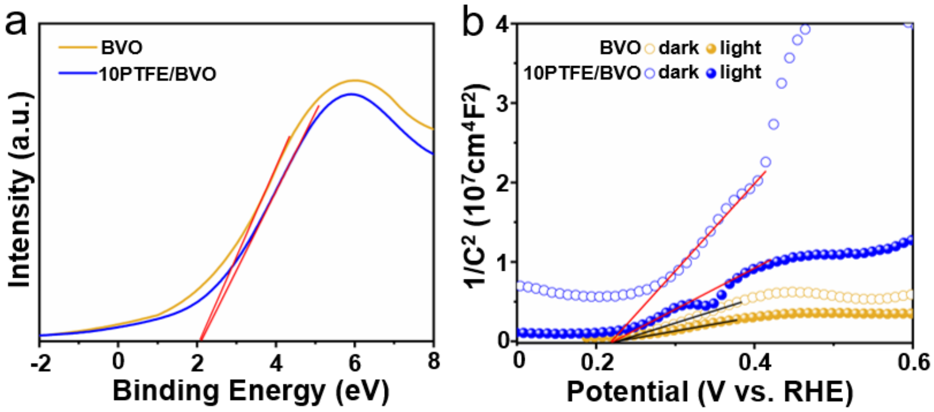

Figure S13. a) VB-XPS spectra of $BiVO_4$ and 10PTFE/BVO, b) Mott-Schottky plots of $BiVO_4$ and 10PTFE/BVO under dark and light illumination in a 1 M $NaHCO_3$ electrolyte.

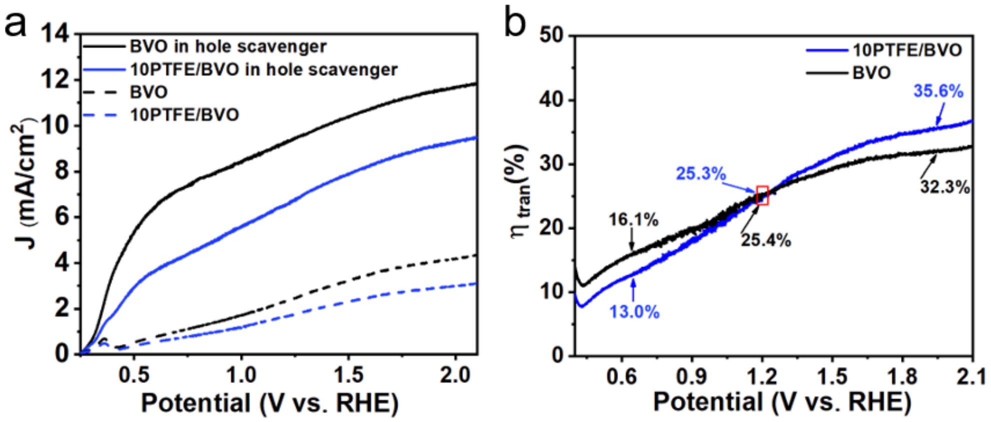

Figure S14 a) Photocurrent density of $BiVO_4$ and 10PTFE/BVO photoanodes with or without holes scavenger (0.5 M Na_2SO_3, 20 mL) in a 1 M $NaHCO_3$ electrolyte, b) interface charge transport and separation efficiencies of $BiVO_4$ and 10PTFE/BVO photoanodes.

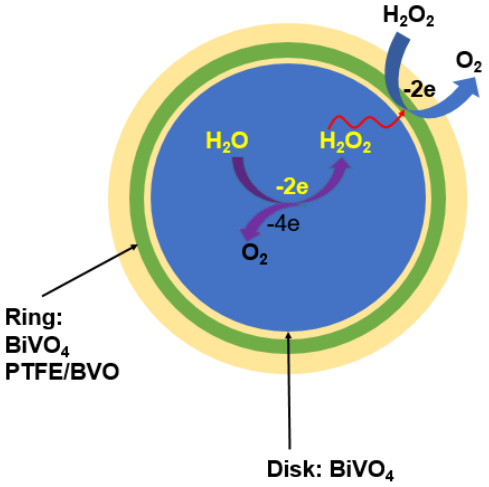

Figure S15. Schematic diagram of the rotating ring-disk electrode system.

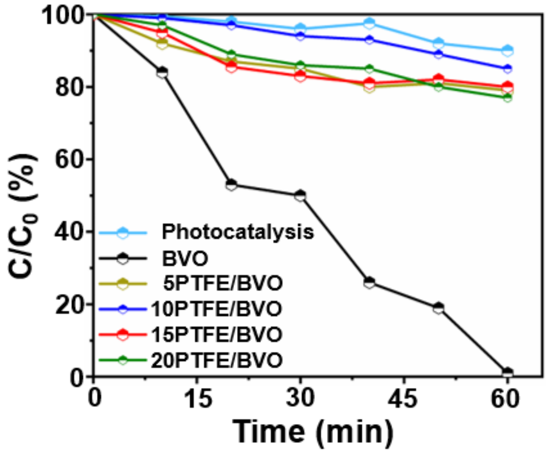

Figure S16. The comparision of photocatalytic H_2O_2 decomposition by $BiVO_4$ and PTFE/$BiVO_4$ with different PTFE coating under light illumination.

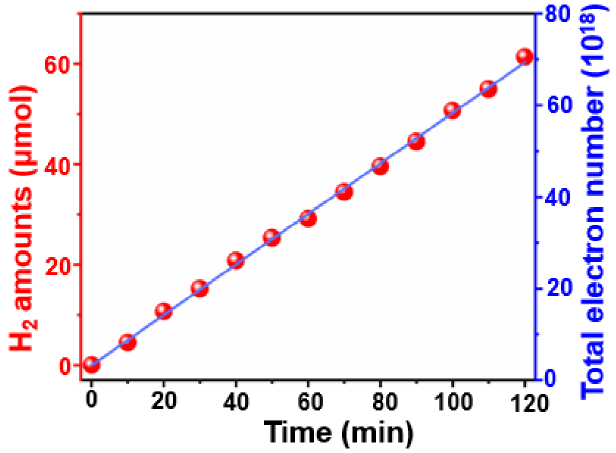

Figure S17. Plots of the theoretical charge number obtained from the J-t curves at 1.23 V vs. RHE and the actual H_2 amounts for 10PTFE/BVO photoanode.

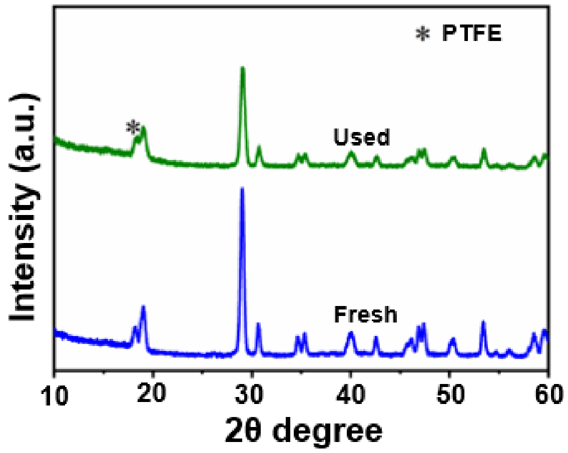

Figure S18. XRD patterns of 10PTFE/BVO photoanode before and after the PEC reaction.

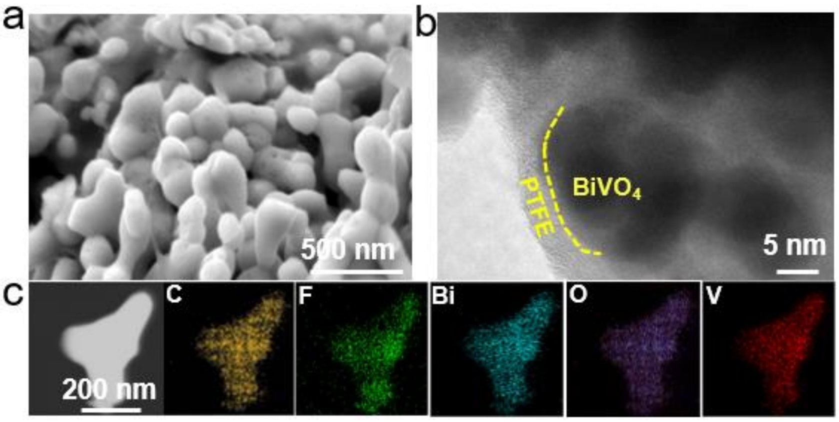

Figure S19. a) op-view SEM, b) HR-TEM and STEM-EDX elemental mapping of the 10PTFE/BVO photoanode after continuous illumination in 1M $NaHCO_3$ electrolyte.

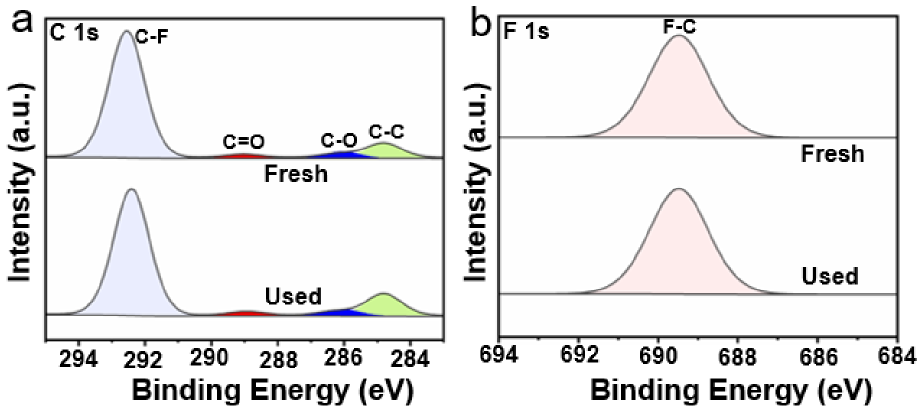

Figure S20. Comparisons of XPS results of 10PTFE/BVO photoanode before and after the PEC reaction: a) C 1s and b) F 1s.

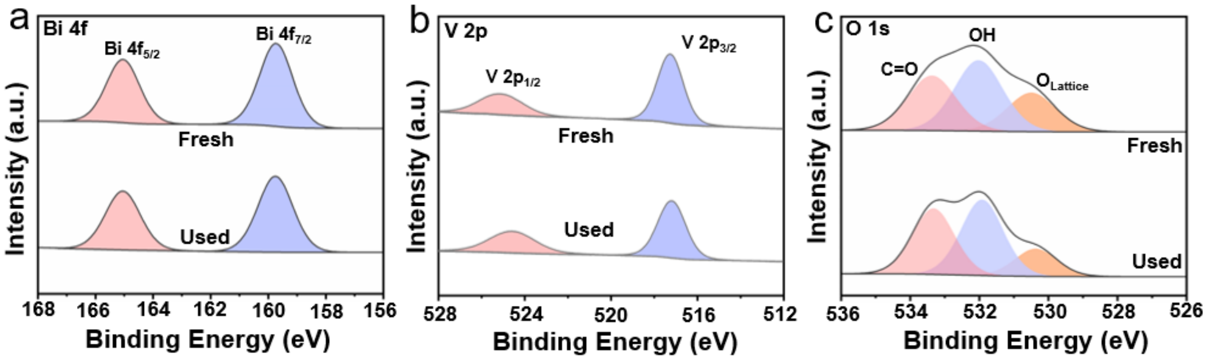

Figure S21. XPS spectra of 10PTFE/BVO before and after the PEC reaction: a) Bi 4f, b) V 2p and c) O 1s.

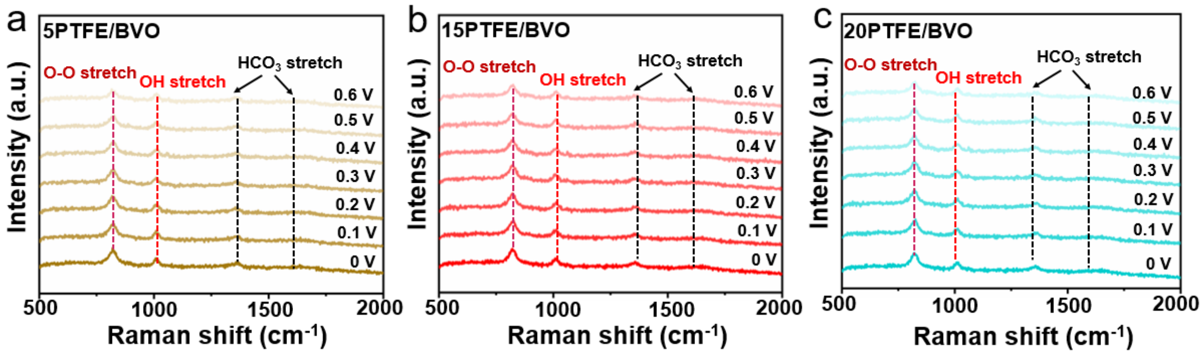

Figure S22. In situ Raman spectra of a) 5PTFE/BVO, b) 15PTFE/BVO and c) 20PTFE/BVO photoanode under different applied biases in 1M $NaHCO_3$ electrolyte. The 0 V means open circuit voltage.

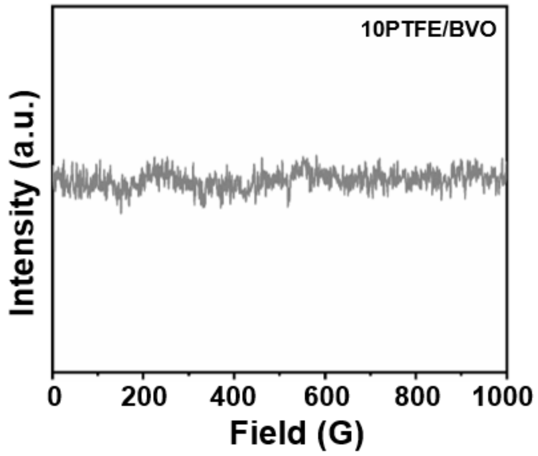

Figure S23. EPR responses of ·OH generation by 10PTFE/BVO photoanode under visible light illumination.

Table S1 Comparison of PCE H_2O_2 generation performance for some representative photoanode under AM 1.5 illumination.

Strategy	Photoanode	Cathode	Electrolyte $KHCO_3$	FE(%)	H_2O_2 accumulation concentration（μmol/L）	Applied Bias	Ref.
Passivation	$Al_2O_3/BiVO_4/WO_3$	Pt	2M	80	120	0.9C	2018
Heterojunction	$WO_3/BiVO_4$	Au	2M	50	80	No bias	2017
	$WO_3/BiVO_4$	Pt	2M	54	220	1.5 V vs RHE	2016
Doping	Mo-$BiVO_4$	C	1M	35	68	1.0 V vs RHE	2020
	W-$BiVO_4$	C	1M	7	10	1.0 V vs RHE	2020
	P-Mo-$BiVO_4$	C	1M	46	105	1.0 V vs RHE	2020
	Cr-$BiVO_4$	C	1M	0.9	2	1.0 V vs RHE	2020
Crystal facets	$BiVO_4$ (010)/(110)	Pt	1M	70	130	0.93 V vs RHE	2021
Microenvironment	PTFE/ $BiVO_4$	Pt	1M	85	150	1.23 V vs RHE	This work

[1] B. K. Trze´sniewski, I. A. Digdaya, T. Nagaki, S. Ravishankar, I. H. -Cardona, D. A. Vermaas, A. Longo, S. Gimenez, W. A. Smith, *Energy Environ Sci* **2017**, *10*, 1517.

[2] a) C. R. Dong, Y. L. Yang, X. M. Hu, Y. J. Cho, G. Y. Jang, Y. H. Ao, L. Y. Wang, J. Y. Shen, J. H. Park, K. Zhang, *Nat Commun* **2022**, *13*, 4982; b) S. Wan, C. Dong, J. Jin, J. Li, Q. Zhong, K. Zhang, J. H. Park, *ACS Energy Lett* **2022**, *7*, 3024.
